# Family, friends, and feelings: the role of relationships to parents and peers and alexithymia in adolescents with anorexia nervosa

**DOI:** 10.1186/s40337-022-00661-3

**Published:** 2022-09-29

**Authors:** Linda Lukas, Christina Buhl, Gerd Schulte-Körne, Anca Sfärlea

**Affiliations:** Department of Child and Adolescent Psychiatry, Psychosomatics and Psychotherapy, University Hospital, LMU Munich, Nussbaumstr. 5, 80336 Munich, Germany

**Keywords:** Anorexia nervosa, Adolescence, Parent relationships, Peer relationships, Alexithymia

## Abstract

**Background:**

Anorexia nervosa (AN) is associated with impairments in socio-emotional functioning, including difficulties in interpersonal relationships as well as alexithymia (difficulties identifying and describing one’s emotions). Although the onset of the disorder is mostly in adolescence, a developmental period in which interpersonal relationships to parents as well as peers undergo major changes, only few studies have investigated the quality of interpersonal relationships in adolescent AN patients. Furthermore, the mechanisms linking poor relationship quality to eating disorder psychopathology are not yet clarified, albeit some research suggests that alexithymia might play a pivotal role. The aims of the present study were investigating the quality of interpersonal relationships to parents and peers in adolescents with AN compared to healthy adolescents as well as exploring the mediating role of alexithymia in the association between relationship quality and eating disorder symptoms.

**Methods:**

Self-report questionnaires were used to assess relationship quality (Inventory of Parent and Peer Attachment) and alexithymia (Toronto Alexithymia Scale) in 12–18 year old female adolescents with AN (*n* = 35) in comparison to healthy adolescents (*n* = 40).

**Results:**

Adolescents with AN reported lower relationship quality to both of their parents and to peers compared to healthy controls. Relationship quality scores were negatively correlated to alexithymia as well as eating disorder symptoms. Alexithymia fully meditated the association between eating disorder symptoms and relationship quality to parents and partially mediated the association between eating disorder symptoms and relationship quality to peers.

**Conclusion:**

The results indicate difficulties in interpersonal relationships among adolescents with AN and emphasize the role of peer relationships for adolescents’ eating disorder psychopathology. Alexithymia seems to play an important role in explaining the link between quality of relationships and eating disorder psychopathology. Results suggest that treatment should not only focus on family relationships but also address relationships to peers as well as adolescents’ competence in identifying and dealing with their emotions.

## Plain English summary

Anorexia nervosa is an eating disorder that affects mostly adolescent and young adult women. Individuals with anorexia nervosa often report problems in their relationships to close persons, e.g., to their parents or peers. Relationships to parents as well as peers undergo major changes during adolescence (with peer relationships gaining major importance), therefore, the present study focused on adolescent girls with anorexia nervosa and assessed the quality of their relationships to parents and peers. As expected, girls with anorexia nervosa reported poorer quality of relationships to their parents and especially to peers compared to healthy girls. Relationship quality was negatively associated with eating disorder symptoms, i.e., the lower relationship quality, the more pronounced were eating disorder symptoms. These results underline that treatment should not only focus on family relationships but also address relationships to peers with therapists encouraging adolescents with anorexia nervosa to establish and maintain friendships. Furthermore, the study revealed that alexithymia (a psychological characteristic involving difficulties in recognising and talking about one’s emotions) plays an important role in explaining the interplay between interpersonal relationships and eating disorder symptoms. However, the direction of effects remains unknown and further studies are needed to elucidate the mechanisms through which problems in relationships with close persons exert their influence on eating disorder symptoms and vice versa.

## Background

Anorexia nervosa (AN) is a mental disorder characterized by significantly low body weight, intense fear of gaining weight and body image disturbance [1]. AN has a lifetime prevalence rate of around 1–2% in women with a peak age of onset between 15 and 19 years of age [2].

In addition to the core symptoms, AN is also associated with problems in socio-emotional functioning [3, 4] which are proposed to contribute to the development and maintenance of the disorder [5–7]. Difficulties in interpersonal relationships may play a particularly important role, since they have been found not only to be associated with eating disorder psychopathology [8] but also to interfere with treatment outcome and completion in eating-disordered individuals [8, 9].

Numerous studies have found women with AN to experience poorer relationships than healthy women. For example, they have been found to report more insecure attachment and to perceive their parents as less caring and more overprotecting in comparison to healthy women (see [4, 8]; for comprehensive reviews and meta-analyses). Insecure attachment seems to be related to eating disorder psychopathology also in adolescence [10], but there are few studies investigating the role of interpersonal relationships in clinical samples of adolescents with AN. This seems of particular relevance not only because adolescence is the most common time for the onset of AN [2], but also because major changes in young people’s relationships take place at this developmental stage [11]: Relationships to parents are subject to major qualitative transformations [12, 13], while relationships to peers gain importance and might become even more influential than parent relationships [14, 15].

The studies investigating attachment and parental relationship quality in adolescents with AN yielded heterogeneous results: Some found no differences in perceived parental care, overprotection, or relationship quality to parents between adolescents with AN and healthy adolescents [16–18], while others found adolescents with AN to report attachment insecurity and lower relationship quality in the relationships to both their mothers and fathers ([19–21]; see [12] for a review). Despite friendships with peers gaining importance in adolescence, only few studies have also investigated peer relationships in adolescents with AN: Two studies found adolescents with AN or other eating disorders to report lower relationship quality not only in their relationships to parents but also to peers in comparison to controls [22, 23] while another study found lower relationship quality in eating disordered adolescents only for relationships to mothers but not peers [24]. Pelletier Brochu et al. [25] found alienation in the relationship to mothers as well as peers, but not fathers, to be positively associated with eating disorder symptom severity in adolescents with AN. The heterogeneous results of these studies underline the need for further research on relationship quality to parents and especially to peers in adolescent AN patients.

What also remains unclear to date are the mechanisms that link insecure attachment and poor relationship quality to eating disorder symptoms [12, 26]. One mechanism that may play an important role is alexithymia, i.e., the inability to recognize and describe one’s own emotions (literally “no words for mood”; [27, 28]). High levels of alexithymia are known to characterize adults [28, 29] as well as adolescents with AN [30–32] and to be positively related to eating disorder symptoms [33, 34]. Alexithymia seems to be associated with relationship quality in young people: Higher levels of alexithymia have been found to be related to lower levels of parental care and support [35, 36] as well as lower relationship quality to and support from peers [35, 37]. Furthermore, alexithymia has been found to be a mediator between relationship quality and different mental health problems such as symptoms of depression and social anxiety [38–40] and has also been suggested to mediate between attachment and aspects of eating disorder psychopathology in adult women [41–43]. Thus, alexithymia might represent a key for understanding the interplay between interpersonal difficulties and eating disorder psychopathology. However, the role of alexithymia in explaining the link between quality of relationships to close individuals and eating disorder symptoms has not been investigated in adolescents with AN to date.

Therefore, the present study was designed to investigate the quality of relationships to parents and peers in adolescents with AN and to explore the role of alexithymia in explaining the link between poor relationship quality and eating disorder psychopathology. The first aim was to investigate the perceived quality of adolescent AN patients’ relationships to parents and peers in comparison to healthy adolescents. In line with previous research, we expected adolescents with AN to report lower quality for all three relationships (i.e., to mothers, fathers, and peers), compared to healthy adolescents. Moreover, we expected relationship quality to be negatively related to eating disorder symptoms. The second aim of our study was to explore the role of alexithymia in explaining this association. We expected alexithymia to be negatively related to relationship quality and positively related to eating disorder symptoms and to mediate the relation between relationship quality and eating disorder symptoms (as suggested by studies in adults with eating disorders).

## Methods

The present data were collected as part of a research project on processing of socio-emotional stimuli in adolescents with AN [44].

### Participants and recruitment

The study sample consisted of 75 adolescent females between the ages of 12 and 18. The *n* = 35 AN patients were recruited at a University Department of Child and Adolescent Psychiatry, Psychosomatics and Psychotherapy in Germany. The *n* = 40 adolescents in the healthy control (HC) group were recruited via previous studies and mailings to randomly-selected families with daughters in the corresponding age range provided by the local registry office.

In all participants, psychiatric diagnoses were assessed using a standardized, semi-structured clinical interview (Kinder-DIPS; [45, 46]) that was conducted and evaluated by trained interviewers. The Kinder-DIPS has been found to show good inter-rater reliabilities for all diagnostic clusters [47] and interrater-reliability in our study was determined for 25% of the sample by an independent researcher rerating audio recordings of the diagnostic interviews. The accordance rate for current diagnosis of AN in the AN group and no lifetime diagnosis in the HC group (predefined criteria) was 100%. Exclusion criteria for all participants were IQ < 85 (measured via the CFT-20-R; [48]), current neurological disorders, pervasive developmental disorders, psychotic disorders, bipolar disorders, and substance abuse.

Adolescents who met DSM-V [1] criteria for AN and had a body mass index (BMI) below the 25th age-corrected percentile^1^ (according to 49) were included in the AN group. Mean illness duration at the time of testing was 22.74 months (*SD* = 23.08; *Mdn* = 17.00; range 1–120). Of the included AN patients, *n* = 16 met criteria for at least one comorbid condition, mostly major depression (*n* = 14, in six of these cases the depressive episode had developed secondary to the eating disorder) and anxiety disorders (*n* = 8).

Adolescents were included in the HC group if they did not meet criteria for any present or past psychiatric disorder as assessed via the Kinder-DIPS.^2^

The study was approved by the institutional ethics committee and all procedures were in accordance with the latest version of the Declaration of Helsinki. Prior to participation, written informed consent was obtained from the participants and their parents/legal custodians after a comprehensive explanation of the study procedures. Participants received a reimbursement of €50 for participation in the whole research project.

### Measures

The perceived quality of interpersonal relationships was measured with the Inventory of Parent and Peer Attachment (IPPA; [50–52]), which has been found to be the best self-report measure to assess attachment/relationship quality in adolescents [53]. We used the German version translated by Rollet et al. [54] which was adapted to correspond to the revised version of the IPPA and to assess relationship quality separately for mothers and fathers, as some of the previous studies suggested that relationships to mothers and fathers might differ [22, 25]. The IPPA assesses adolescents’ evaluation of the quality of their relationships to their mother, father, and peers (i.e., “friends”) on three subscales: Trust, Communication, and Alienation. The Trust subscale assesses the degree of mutual understanding and respect, the Communication subscale measures the extent and quality of spoken communication, and the Alienation subscale captures feelings of anger and detachment [52]. The negative subscale Alienation was inverted to enable the calculation of total relationship quality scores across subscales [51]. Each of the three sections (mother, father, peers) consisted of 25 items that were rated on a 5-point scale. IPPA scores were available for 73 (mother section), 72 (father section), and 74 (peer section) of the 75 participants. Internal consistencies in our study were excellent for the total relationship quality scores for mothers, fathers, and peers (Cronbach’s αs ranging between .94 and .96) and at least good for the subscales Trust, Communication, and Alienation within the sections (Cronbach’s αs ranging between .80 and .95).

Alexithymia was assessed with the German version of the Toronto Alexithymia Scale (TAS; [55]). The TAS is a self-report questionnaire assessing alexithymia on three subscales: Difficulty Identifying Feelings, Difficulty Describing Feelings, and Externally Oriented Thinking. However, as previous studies have found the Externally Oriented Thinking subscale to have poor psychometric properties^3^ in adolescent samples and suggested the Difficulty Identifying Feelings and Difficulty Describing Feelings subscales to represent the core dimensions of alexithymia [38, 56], only these subscales were analysed in the present study and summed up to a total alexithymia score (cf. [38, 39]). TAS scores were available for all participants. Internal consistency for the TAS total score was good in our sample (Cronbach’s α = .90).

In addition, we assessed clinical characteristics of our samples. For participants in the AN group, height and weight were obtained from their physicians while for participants in the HC group, height and weight were measured in our laboratory. Eating disorder symptom severity was measured with the German version of Eating Disorder Inventory 2 (EDI; [57]) and depressive symptoms were assessed with the German version of the Beck Depression Inventory-II (BDI; [58]). Both instruments allow a valid assessment of the respective symptoms in adolescent samples [59, 60]. EDI scores were available for 74 of the 75 participants and BDI scores were available for all participants. Internal consistencies in our sample were excellent (EDI total score: Cronbach’s α = .98; BDI: Cronbach’s α = .97).

### Data analysis

Statistical analyses were performed with SPSS. Separate multivariate analyses of variance (MANOVAs) were conducted to assess group differences in relationship quality to mothers, fathers, and peers with Group as the independent variable and the relationship quality subscales (Trust, Communication, Alienation) as dependent variables.^4^ Pearson correlations were calculated across groups to examine relations between relationship quality, alexithymia, and eating disorder symptoms. Furthermore, mediation analyses were conducted to assess if alexithymia mediates the relationships between relationship quality to mothers, fathers, and peers and eating disorder symptoms. Mediation analyses were performed using the PROCESS macro for SPSS (v.3.5; [61]). Bootstrapping with 5000 samples and heteroscedasticity consistent standard errors were employed to compute the confidence intervals (CI) and inferential statistics. Indirect effects were considered significant if the 95% CI did not include zero. For all other effects, the level of significance was set to *p* = .05 (two-tailed).

## Results

### Sample characteristics

The characteristics of the two groups are presented in Table 1. As expected, the groups did not differ regarding age and IQ but regarding eating disorder related variables (BMI and BMI-percentile, eating disorder symptoms) as well as depressive symptoms and alexithymia.

**Table 1:** Characteristics of the study sample

	AN	HC	*t*	*p*
	*n* = 35	*n* = 40		
	*M (SD)*	*M (SD)*		
Age	15.74 (1.70)	15.41 (1.64)	< 1	n.s
IQ	105.94 (12.65)	105.78 (12.20)	< 1	n.s
BMI	15.52 (1.61)	20.39 (2.26)	10.84	< .001
BMI-percentile (age-corrected)	3.43 (4.95)	49.53 (25.22)	11.31	< .001
Depressive symptoms (BDI)	25.09 (14.54)	2.55 (3.30)	10.25	< .001
Eating disorder symptoms (EDI)	308.59 (61.80)	182.28 (39.70)	8.97	< .001
Alexithymia total (TAS)	35.34 (8.12)	23.68 (7.84)	6.32	< .001

*AN* anorexia nervosa, *HC* healthy control, *IQ* intelligence quotient, *BMI* body mass index, *BDI* Beck Depression Inventory, *EDI* Eating Disorder Inventory, *TAS* Toronto Alexithymia Scale, *M* mean, *SD* standard deviation

### Group comparisons regarding relationship quality

Descriptive data regarding relationship quality can be found in Table 2. Regarding the relationship to mothers, the MANOVA indicated an overall difference between AN and HC group, (V = .23, *F*_3,69_ = 6.89, *p* < .001, *η*_*p*_^*2*^ = .23). Separate univariate ANOVAs on the outcome variables showed that the groups differed significantly on all subscales: Trust (*F*_1,71_ = 16.00, *p* < .001), Communication (*F*_1,71_ = 15.28; *p* < .001), and Alienation (*F*_1,71_ = 16.28, *p* < .001).

**Table 2:** Relationship quality in AN versus HC group

	AN	HC	*p*	*d*
	*MD (SD)*	*MD (SD)*		
**Relationship quality mothers**	93.34 (14.79)	108.24 (12.84)	< .001	1.08
Trust	40.97 (7.50)	46.54 (3.80)	< .001	0.94
Communication	31.69 (4.94)	37.11 (6.70)	< .001	0.92
Alienation	20.69 (4.34)	24.66 (4.06)	< .001	0.94
**Relationship quality fathers**	86.18 (18.78)	99.16 (20.36)	.003	0.66
Trust	39.15 (7.62)	43.71 (6.92)	.010	0.63
Communication	27.75 (7.78)	31.97 (9.81)	.049	0.48
Alienation	19.28 (4.32)	23.47 (5.15)	< .001	0.88
**Relationship quality peers**	84.76 (18.60)	108.98 (10.48)	< .001	1.60
Trust	37.65 (8.91)	47.23 (3.77)	< .001	1.40
Communication	25.87 (6.79)	33.90 (4.50)	< .001	1.39
Alienation	21.25 (4.69)	27.85 (4.05)	< .001	1.51

*AN* anorexia nervosa, *HC* healthy control, *M* mean, *SD* standard deviation

Regarding the relationship to fathers, the MANOVA revealed an overall difference between the groups (V = .18, *F*_3,68_ = 5.10, *p* = .003, *η*_*p*_^*2*^ = .18). Again, univariate ANOVAs indicated group differences in all subscales (Trust: *F*_1,70_ = 7.09, *p* = .010): Communication: *F*_1,70_ = 4.03, *p* = .049; Alienation: *F*_1,70_ = 13.84, *p* < .001).

With respect to the relationship to peers, the MANOVA indicated an overall difference between groups (V = .42, *F*_3,70_ = 16.58, *p* < .001, *η*_*p*_^*2*^ = 0=.42). Univariate ANOVAs revealed significant group differences on all three subscales (Trust: *F*_1,72_ = 38.24, *p* < .001; Communication: *F*_1,72_ = 36.91, *p* < .001; Alienation: *F*_1,72_ = 42.13, *p* < .001).

For all three relationships, AN patients reported lower trust and communication as well as higher alienation^5^ compared to the HC group. Effect sizes for the group differences were medium to large for relationship quality to fathers and large regarding relationships to mothers and peers, with largest effect sizes for relationships to peers (see Table 2).

### Correlations

Relationship quality was negatively correlated with alexithymia as well as eating disorder symptoms (Table 3). Correlations were moderate to strong with strongest correlations found for relationship quality to peers (significantly larger than correlations between relationships to parents and eating disorder symptoms: *z*s ≥ 2.25, *p*s ≤ .025; [62]). Alexithymia and eating disorder symptoms were strongly positively correlated.

**Table 3:** Pearson correlations

	Relationship quality mothers	Relationship quality fathers	Relationship quality peers	Alexithymia total (TAS)
Relationship quality fathers	.59***			
Relationship quality peers	.41***	.41***		
Alexithymia total (TAS)	− .41**	− .46***	− .62***	
Eating disorder symptoms (EDI)	− .49***	− .47***	− .70***	.80***

*EDI* Eating Disorder Inventory, *TAS* Toronto Alexithymia Scale

**p < .01; ***p < .001

### Mediation analyses

**Fig. 1:**
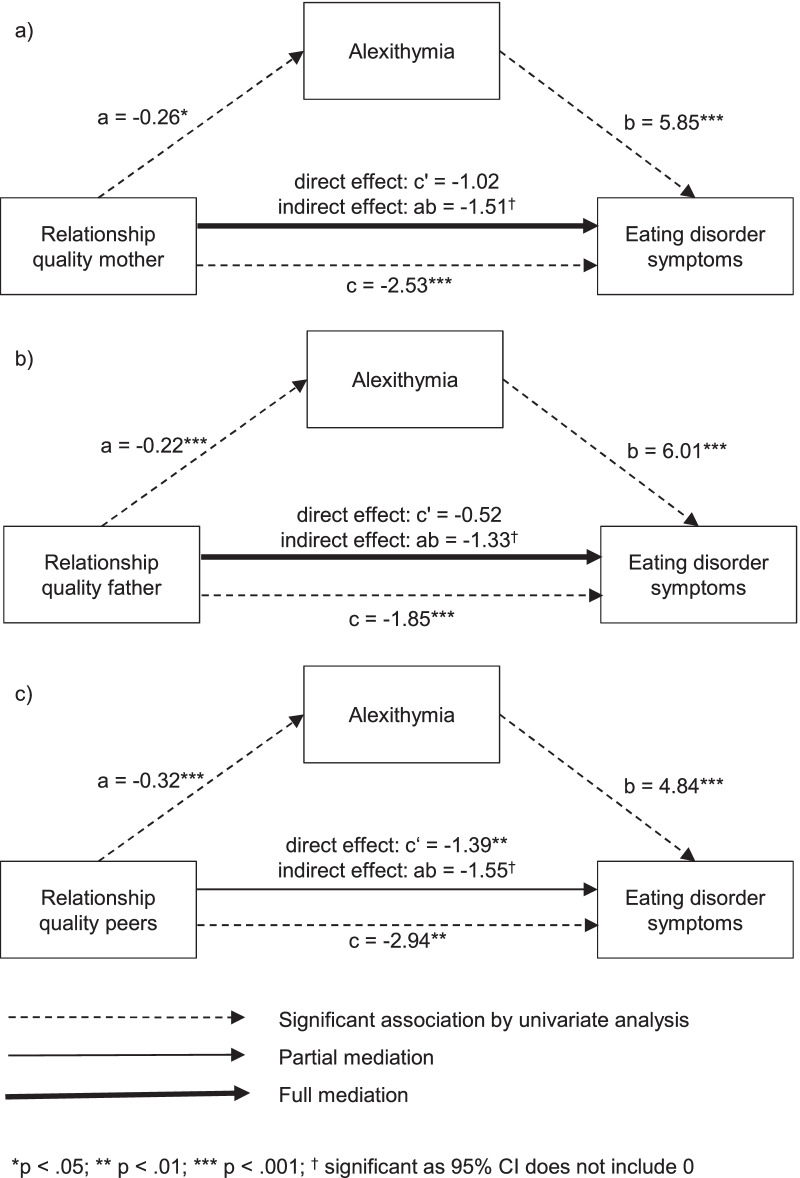
Results of the mediation analyses of alexithymia mediating relations between relationship quality to mothers (**a**), fathers (**b**), and peers (**c**) and eating disorder symptoms

Mediation analyses are graphically summarized in Fig. 1. The mediation analysis with relationship quality to mothers as predictor, eating disorder symptoms as outcome, and alexithymia as mediator revealed a significant total effect (*b* = − 2.53, *p* < .001). Relationship quality to mothers significantly predicted alexithymia (*b* = − 0.26, *p* = .018) which in turn significantly predicted eating disorder symptoms (*b* = 5.85, *p* < .001). Alexithymia fully mediated the relation between relationship quality to mothers and eating disorder symptoms (direct effect non-significant: *b* = − 1.02, *p* = .062; indirect effect: *b* = − 1.51, 95% CI [− 3.01, − 0.64], κ^2^ = − .29 [− .48, − .14]^6^).

The mediation analysis with relationship quality to fathers as predictor revealed a significant total effect (*b* = − 1.85, *p* < .001). Relationship quality to fathers significantly predicted alexithymia (*b* = − 0.22, *p* < .001) which in turn significantly predicted eating disorder symptoms (*b* = 6.01, *p* < .001). Alexithymia fully mediated the relation between relationship quality to fathers and eating disorder symptoms (direct effect non-significant: *b* = − 0.52, *p* = .230; indirect effect: *b* = − 1.33, 95%-CI [− 2.18, − 0.71], κ^2^ = .34 [− .49, − .20]^6^).

The mediation analysis with relationship quality to peers as predictor revealed a significant total effect (*b* = − 2.94, *p* < .001). Relationship quality to peers significantly predicted alexithymia (*b* = − 0.32, *p* < .001) which in turn significantly predicted eating disorder symptoms (*b* = 4.84, *p* < .001). Alexithymia partially mediated the relation between relationship quality to peers and eating disorder symptoms (direct effect: *b* = − 1.39, *p* = .004; indirect effect: *b* = − 1.55, 95%-CI [− 2.28, − 0.95], κ^2^ = − .37 [− .50, − .24]^6^).

In summary, all indirect effects of relationship quality on eating disorder symptoms through alexithymia were strong. Between relationship quality to peers and eating disorder symptoms an additional direct effect emerged. The mediation analyses were repeated with diagnosis of AN as outcome instead of eating disorder symptoms, yielding a similar pattern of results.

## Discussion

The present study investigated relationship quality to mothers, fathers, and peers in adolescents with AN and healthy adolescents and explored the role of alexithymia in explaining the link between relationship quality and eating disorder symptoms. Adolescents with AN reported lower quality of relationships than healthy adolescents for all examined relationships. Relationship quality scores were negatively correlated to alexithymia as well as eating disorder symptoms. Alexithymia mediated the association between relationship quality to parents and eating disorder symptoms fully and between relationship quality to peers and eating disorder symptoms partially.

In line with our expectations and previous literature (e.g., [21, 22]; but see also [18]), we found adolescents with AN to report lower quality for all three relationships, i.e., to mothers, fathers, and peers, compared to healthy adolescents. The groups differed not only in all three relationships but also on all subscales of relationship quality: adolescents with AN reported lower Trust, lower Communication, and higher Alienation in their relationships than healthy adolescents. In addition, relationship quality scores were negatively correlated with eating disorder symptoms (in line with [19, 23, 25]). Effect sizes for group differences were largest for relationships to peers and the correlation with eating disorder symptoms was significantly larger for relationships to peers than for relationships to parents, suggesting that peer relationships might be particularly impaired in adolescents with AN. Previous studies investigating the relative influence of parent and peer relationships on adolescent mental health yielded heterogeneous results: while, e.g., Laible et al. [14] found peer relationships to be more influential, others [39] found parent relationships to be more closely related to adolescents' mental health. Our study suggests that for eating disorder psychopathology friendships to peers play a particularly important role. This is in line with Cunha et al. [22] but contrasts the results of others [23, 25] who found eating disorder psychopathology to be related more strongly to relationship quality to mothers and/or fathers than to relationship quality to peers.

The second aim of our study was to explore the role of alexithymia in explaining the association between relationship quality and eating disorder symptoms. As expected, adolescents with AN showed higher alexithymia scores than healthy adolescents (in line with [30–33, 34]) and alexithymia scores were negatively related to relationship quality [39, 41] and positively related to eating disorder symptoms [33, 34], i.e., the higher alexithymia scores were, the lower were relationship quality scores and the higher were eating disorder symptoms. Alexithymia turned out to be a full mediator for the associations between relationship quality to mothers and fathers and eating disorder symptoms. This suggests that indeed alexithymia is a mechanism that links parent relationship quality to eating disorder symptoms in adolescents. Parents represent an influential source for learning how to identify, interpret, and label emotions [64, 65], so individuals with poor relationships to parents presumably have less opportunities to develop accurate representations of their affective states, leading to alexithymia [42, 43, 64]. The inability to reflect upon one’s feelings may cause discomfort and may also impede the ability to generate appropriate reactions to one’s feelings (i.e., adaptive emotion regulation; [30]). Eating disorder symptoms could then arise as an attempt to counteract the discomfort [26] or as a maladaptive way to regulate or escape from negative emotions [26, 66], for example by distraction by weight-loss behaviors or by feeling “less” in the state of starvation.

The association between relationship quality to peers and eating disorder symptoms was only partially mediated by alexithymia: A significant direct effect from peer relationship quality to eating disorder symptoms was present in addition to the indirect effect through alexithymia. This means that the link between peer relationship quality and eating disorder symptoms cannot be fully explained by alexithymia. Maybe this is because peer relationships not only influence alexithymia but are also influenced by alexithymia: Alexithymia may not only result from inadequate relationships, especially to parents [64], but may also lead to difficulties in establishing and sustaining friendships with peers, as the inability to recognize and communicate one’s feelings interferes with intimacy within relationships and might contribute to individuals being perceived as emotionally distant and withdrawn [39]. This lack of good friendships with peers might then influence eating disorder psychopathology in different ways: (1) peers have been suggested to be an important source for “normative” standards, especially regarding appearance [23, 67] and the lack of the normalizing influence^7^ of the peer group might lead to a greater endorsement of problematic eating attitudes and behaviors in adolescents [25, 70], (2) poor peer relationships might involve teasing about weight and physical appearance, which has been found to be a predictor for unhealthy eating attitudes and behaviors [71], (3) relatedly, difficulties in peer relations might lead adolescents to engage in disordered eating behaviors as an attempt to gain social approval and acceptance [67], and (4) importantly, social isolation and the lack of good friendships could cause or be accompanied by low self-esteem or depressive symptoms [25, 67, 72] that might trigger disordered eating behavior as a means to either achieve positive self-worth or deal with negative affect [25, 66, 72]. These multiple pathways might explain the particularly important role that peer relationships seem to play for eating disorder psychopathology.

Importantly, due to the cross-sectional design of our study, our results do not imply any causality and do not allow statements about the direction of effects. It remains unknown to what extent the quality of interpersonal relationships in adolescents with AN has been poor before disorder onset and to what extent it decreased as a result of the disorder. Individuals with AN have been found to engage less in social activities even before disorder onset [73] and poor friendship quality has been found to be related to greater eating pathology also in non-selected samples of adolescent females [67, 72], suggesting that a lack of social support might place adolescents at risk for the development of AN. On the other hand, Patel and colleagues [74] found adolescents with AN to report dissolving friendships and an impoverished social network as a consequence of their disorder. Westwood et al. [75] as well as Cardi et al. [76] found that many individuals with AN reported having had social difficulties before disorder onset, but even more reported that the disorder had affected their relationships in a negative way. It is possible that eating disturbances interfere with adolescents’ abilities to sustain healthy relationships to friends [67], for example due to avoidance of eating-related activities or time-consuming weight-loss behaviors like excessive exercising. Relationships to parents could also be adversely affected by the eating disorder, with trust dwindling, alienation increasing, and communication deteriorating as a consequence of recurring familial arguments about food intake or weight-loss behaviors. Hence, the connection between relationship quality and AN is likely to be bidirectional with premorbidly poorer social relationships further deteriorating as a consequence of the disorder. Regarding alexithymia, it has already been delineated that its association with relationship quality is likely to be bidirectional. This might also be the case for the association between alexithymia and eating disorder symptoms: It is possible that alexithymia is not a premorbid trait that places individuals at risk for developing AN but rather a state that manifests as a byproduct of the eating disorder (for example due to suppression of unpleasant cognitions and emotions related to eating or body weight and shape; [43]). Thus, all mechanisms suggested above remain hypothetical. It also has to be noted that relationship quality scores to mothers, fathers, and peers are not independent from each other: There was a large correlation between relationship quality to mothers and fathers and medium correlations between relationship quality to peers and parents. This is in line with the finding that different types of relationships in adolescents are distinct but at the same time related, presumably because parent relationships shape other close relationships like those to peers [77]. It suggests, with regard to our study, that the influence of the different relationships on alexithymia and eating disorder symptoms might be interdependent. Studies in larger samples that allow using structural equation modeling along with longitudinal studies are essential to shed proper light on the proposed pathways and disentangle the interplay between relationship quality, alexithymia, and eating disorder symptoms.

Further limitations also need to be considered. It is not known to what extent our results are specific for AN: A relatively high proportion of adolescents in our AN group also met criteria for comorbid disorders, mostly major depression (40%) or anxiety disorders (23%). Even though this is expected [78], it makes it impossible to isolate the specific contribution of eating disorder psychopathology. Some previous studies suggested that increased alexithymia [30] and difficulties in relationships with parents [79, 80] might be transdiagnostic characteristics rather than specific attributes of adolescents with AN. It is possible, for example, that depressive symptoms contribute to a negative view of the quality of social relationships [81, 82] and explain increased alexithymia [83, 84], thus playing an important role for all hypothesized mechanisms. However, depressive symptoms and eating disorder symptoms were highly correlated in our sample, not only across groups but also within AN and HC groups (*r*s ≥ .52), suggesting that eating disorder and depressive psychopathology can hardly be disentangled and trying to artificially remove variance explained by depressive symptoms is not reasonable. Future studies need to address this limitation by including clinical control groups that show similar depression and anxiety symptoms as adolescents with AN but no eating disorder psychopathology.

Future studies on relationship quality in adolescents with AN may also consider investigating not only relationships to parents and peers but also to siblings, as individuals with AN often feel isolated and misunderstood by their siblings [85] and their relationships to siblings are also adversely affected by the disorder [86, 87].

The close connection of relationship quality and eating disorder psychopathology underlines the importance of addressing interpersonal relationships in the treatment of adolescents with AN [7, 70, 88]. Importantly, according to our results peer relationships seem to play a particularly important role, suggesting that treatment should not only focus on family relationships but also address friendships to peers, which have been neglected not only by research but also by therapy to date [89]. It has been suggested that the maintenance of peer relationships during and after treatment is crucial for adolescents with AN in order to sustain a sense of normality [90] and that the magnitude and quality of peer support has an important impact on how adolescents’ treatment progresses and how they experience their lives after treatment [91]. Therefore, therapists should encourage adolescents to maintain and establish friendships and help them identify and overcome barriers for doing so. In addition, the substantial role alexithymia seems to have in the interplay between relationship quality and eating disorder psychopathology (although the direction of effects remains unclear), together with the finding that alexithymia has a negative impact on treatment outcome in patients with eating disorders [92], suggests that adolescents with AN are likely to benefit from interventions addressing their alexithymia. Learning how to identify and describe their emotions might not only improve their emotion regulation abilities [30, 93] but might also help them to build stable and functional relationships with parents and peers, thereby alleviating the detrimental influence of poor interpersonal relationships on the maintenance of eating disorder psychopathology.

## Conclusion

The present study is one of few investigating relationship quality not only to parents but also to peers in adolescents with AN. Our results emphasize the role of peer relationships for adolescents’ eating disorder psychopathology and suggest that treatment should not only focus on family relationships but also address relationships to peers. Furthermore, our results revealed that alexithymia plays an important role in explaining the link between quality of relationships and eating disorder psychopathology. However, as the direction of effects and causal mechanisms remain unclear, further studies are needed to elucidate possible pathways.

## Data Availability

The data are available from the corresponding author on reasonable request.

## Abbreviations

AN: Anorexia nervosa
BDI: Beck depression inventory-II
BMI: Body mass index
EDI: Eating disorder inventory 2
HC: Healthy control
IPPA: Inventory of Parent and Peer Attachment
Kinder-DIPS: Diagnostisches Interview bei psychischen Störungen im Kindes- und Jugendalter [Diagnostic interview for mental disorders in childhood and adolescence]
MANOVA: Multivariate analysis of variance
TAS: Toronto Alexithymia Scale

## Footnotes

1 We chose below the 25th percentile as inclusion criterion as this allowed us to include participants with atypical AN, i.e., females who were not underweight (yet) but had lost a significant amount of weight through unhealthy behaviors and had pronounced AN symptoms as weight phobia and body image disturbance, and b) participants who had been underweight but had already gained some weight in the inpatient setting (i.e., were partly weight-restored) but still showed pronounced AN symptoms as weight phobia and body image disturbance. Therefore, two participants in our AN group had a BMI above the 10^th^ percentile. To examine if these participants influenced our results, we repeated all analyses were excluding them. The pattern of results remained the same, therefore the results based on the whole sample are reported.

2 After the diagnostic interview *n* = 3 females that were recruited for the HC group could not be included due to (a history of) psychiatric disorders and *n* = 2 females recruited for the AN group could not be included due to not meeting criteria for AN. One further HC participant could not be included in the present study as she did not fill out any of the relevant questionnaires. This resulted in the above described study sample of *N* = 75.

3 Internal consistency in our sample was also unacceptable: Cronbach’s α = .51.

4 These analyses were performed as MANOVAs with relationship quality subscales as separate dependent variables as previous research indicated that Alienation might play a particularly important role in adolescents with AN [22, 25]. However, as our results indicated that groups differed in all subscales (see Results section) and subscales were highly correlated within the sections (*r*s ≥ .63), we decided to run further analyses using the total scores for relationship quality to mothers, fathers, and peers.

5 As the Alienation subscale was inverted, lower scores on this scale indicate more pronounced alienation.

6 The effect size κ^2^ ranges from 0 to 1. A value of .01 can be interpreted as a small effect, .09 is a medium effect and .25 represents a large effect [63].

7 It has to be noted that standards set by peers and physical appearance comparison with one’s peer group can also have a detrimental influence on the development of body dissatisfaction and disordered eating [68, 69]. Peer experiences provide an important social context during adolescence in which standards of appearance and ideals are modeled and reinforced. So on the one hand, dysfunctional attitudes and assumption may be reinforced by peers, fostering eating disordered behavior, while on the other hand, dysfunctional attitudes and assumption may be “corrected” through peers’ more healthy attitudes towards appearance and eating. The quality of peer relationships might make a difference, with better relationships exerting a more beneficial influence, but investigating this was beyond the scope of the present study and remains the subject of future research.
